# Psychiatry

**Published:** 1885-12

**Authors:** 


**Psychiatry.** A clinical treatise on diseases of the fore brain, based upon a study of its structures, functions and nutrition. By THEODOR MEYNERT, M. D., Professor of Nervous Diseases, and Chief of the Psychiatric Clinic in Vienna. Translated by B. Sachs, M. D., Instructor in Diseases of the Mind and Nervous System in the New York Polyclinic. Part I. The Anatomy, Physiology and Chemistry of the Brain. New York and London: G. P. Putnam's Sons. 1885.

"It is a scientific treatise on diseases of the mind by the one best fitted to write such a treatise. To most medical men, Meynert is known as the great brain-anatomist." There is no question but that the two foregoing statements by the translator are true. There is no work in any language more complete, and any physician who reads only English will feel grateful to Dr. Sachs for the translation of this most excellent book. The translation, while it is "not literal," gives the exact meaning of the original in all parts. To those alone who have translated German, the arduous task performed in the translation of such a work as this becomes apparent. The translation is good. The work is illustrated with many cuts of the brain and spinal cord; they are all excellent. The printing and general style of the work is pleasing. Many will await anxiously the second edition of this work. We bespeak for this book, on an interesting and growing subject, a large sale.

